# Siji Antiviral Mixture Protects against CA16 Induced Brain Injury through Inhibiting PERK/STAT3/NF-*κ*B Pathway

**DOI:** 10.1155/2018/8475463

**Published:** 2018-08-16

**Authors:** Huimin Xiao, Rong Zhao, Yue He, Yang Liu, Qiaoyan He, Siwang Wang, Fei Chen

**Affiliations:** ^1^Department of Chinese Materia Medica and Natural Medicines, Air Force Medical University, No. 169, West Changle Road, Xi'an 710032, China; ^2^Reseach and Development Department, Shaanxi Haitian Pharmaceutical Limited Company, Xianyang 712000, China; ^3^College of Chemistry and Pharmacy, North West Agriculture and Forestry University, Xi'an 710000, China; ^4^Department of General Surgery, Wuhan No. 1 Hospital, No. 215 Zhongshan Road, Wuhan 43000, China

## Abstract

Coxsackievirus 16 (CA16) causes hand, foot, and mouth disease (HFMD) in young children and infants, and it can lead to fatal neurological complications. This study investigated antiviral effects of Siji Antiviral Mixture (SAM) on CA16 in neonatal mice and the protective effects of SAM on CA16 induced brain injuries. Neonatal BALB/c mice and SH-SY5Y cells were used and injected with CA16 stains to study the efficacy. ELISA and Western blotting were used to measure the cytokines levels and proteins expression. Genes transduction was also used to verify interaction mechanism. As the results shown, SAM could reduce the clinical scores at the beginning and delay disease development* in vivo*. Treatment with SAM decreased the levels of LDH, CK-MB, caspase 3 and Bax, ER stress, and inflammatory reaction induced by CA16 infection. Further siRNA transfection results showed that CA16 induced ER stress and inflammatory reaction through PERK/STAT3/NF-*κ*B signaling and the protective effects of SAM might be through inhibiting PERK/STAT3/NF-*κ*B signaling. HPLC analysis showed fingerprint profiles of SAM had 42 chromatographic peaks. Collectively, our study highlighted distinct roles of SAM in inhibiting CA16 infection and brain injury. The molecular mechanism of SAM might be through inhibiting PERK/STAT3/NF-*κ*B signaling.

## 1. Introduction

Hand, foot, and mouth disease (HFMD), caused by serotypes of the* Enterovirus A* species, is a common childhood illness in department of pediatrics [1, 2]. HFMD was first reported in Toronto, Canada, in 1957, and it then outbroke around the world [3–6]. An outbreak of HFMD in China was first reported in 1981 in Shanghai. From May 2008 to December 2009, more than 1065000 cases of HFMD were reported in Mainland China and the disease was relatively frequent in Beijing city, Zhejiang province, Shanghai city, and Hainan province [7]. HFMD has been classified by the Chinese government as a C-class notifiable disease from 2008 in China. The annual cumulative total of reported cases in China had increased from 1 million in 2008 to 7 million cases in 2014 [8]. Another literature also reported that the morbidity of HFMD in China increased from 37.6/100 000 in 2008 to 139.6/100 000 in 2014 [9]. Thus, HFMD has been a growing public health concern and been a considerable economic burden and health impact in affected areas. However, there was no vaccine or antiviral efficient drug to combat HFMD. Antiviral agent ribavirin and immunoglobulin are commonly used clinically, but the therapeutic effect remains uncertain [10, 11].

Human Enterovirus 71 (EV-A71) and Coxsackievirus 16 (CV-A16) are two main etiologic agents, which share some common structural characteristics and belong to the Enterovirus genus of the Picornaviridae family [12, 13]. In HFMD, several complications were reported, including meningitis, encephalitis, acute cardiopulmonary failure, and other organ injury [14, 15]. These complications may result in significant morbidity or even mortality. A study about the distribution of enteroviruses in hospitalized children with HFMD showed that about 21% were EV71 infection and 16.1% were CA16 infection. And they also found that 70.7% of patients with nervous system damage were EV71 infection, and 20.6% were CA16 infection [16]. Compared with the EV71 infection, neurological injuries resulting from CA16 infection are mild and deaths are especially rare. Therefore, CA16 infection has attracted little attention [17–19]. However, we found that CA16 can also cause severe neurological symptoms in HFMD. Thus, research and development of antiviral drugs which prevent CVA16 infections and treat HFMD related neurovascular complications must be an effective way.

Traditional medicinal plants might be a suitable alternative to antiviral drugs. Some medicinal plants and preparations containing plants extracts have been proven to be of therapeutic efficacy against HFMD in China [20]. They were also defined as traditional Chinese medicine (TCM). Some TCM have been shown to ameliorate mild fever, rash, and some other symptoms, and others to treat the complications [21]. Compared with some antiviral drugs, TCMs share some common features including minimal side effects, low potential to cause resistance, multiple targets, and being cheaper. Screening of the commonly used medicinal plants for HFMD therapy in China has led to the discovery of potent efficacy of the antiviral and anti-inflammatory activity [20].

Siji Antiviral Mixture (SAM), a compound of Chinese medicine, has been approved by China State Food and Drug Administration and used for the treatment of viral cold, influenza, mumps, and other virus infectious diseases. In recent years, SAM was also used to treat pediatric respiratory infection and HFMD [22]. The active components of SAM are extracted from* H. cordata*,* platycodon root*,* mulberry leaf*,* forsythia*,* herba schizonepetae*,* mint*,* purple Perilla leaf*,* bitter almond, phragmites communis, chrysanthemum*, and* licorice*. However, the therapeutic mechanism of SAM against CA16 infection and associated neurovascular complication still remains unclear. This study was designed to verify anti-infective effect of SAM during HFMD and find the potential mechanisms.

## 2. Materials and Methods

### 2.1. Reagents

Siji Antiviral Mixture (SAM, 0.97g/mL, batch number: 20150904) was obtained from Shaanxi Haitian Pharmaceutical Co., Ltd., which was prepared from water and ethanol extracts of* H. cordata*,* platycodon root*,* mulberry leaf*,* forsythia*,* herba schizonepetae*,* mint*,* purple Perilla leaf*,* bitter almond, phragmites communis, chrysanthemum*, and* licorice *according to the guidelines of Good Manufacturing Practice and Good Laboratory Practice. Hospital agency and Shaanxi provincial food and drug administration determined the content of its major components. Saline was used dissolve SAM.

Kits for interleukin-6 (IL-6), chemokine (C-C motif) ligand 2 (CCL2), Cyclooxygenase 2 (COX-2), and caspase 3 detection were purchased from Nanjing Jiancheng Bioengineering Institute (Nanjing, China). PERK lentiviral activation particles and PERK, STAT3, and NF-kB siRNA plasmids were obtained from (Santa Cruz, CA, USA). The antibodies against STAT3, PERK, P-PERK, elF2*α*, P-elF2*α*, NF-kB, GAPDH, OASIS, and CHOP were obtained from Santa Cruz Biotechnology (Santa Cruz, CA, USA). The antibodies against cleaved-caspase 3, Bax, and Bcl-2 were obtained from Sigma-Aldrich (St Louis, MO). Other reagents were from Sigma-Aldrich.

### 2.2. Virus and Cells

The CV16 stain was obtained from clinical isolates in the Department of Pediatrics at the Xijing Hospital at Xi'an, China. The SH-SY5Y human neuroblastoma cell line was purchased from the Cell Bank of Chinese Academy Sciences (Shanghai, China). The cells were maintained in Dulbecco's modified Eagle's medium (DMEM) supplemented with 10% fetal bovine serum (FBS), streptomycin (100 U/ml), penicillin (100 U/ml), and 2 mM L-glutamine (all purchased from Life Technologies, USA). Cells were cultured in a humidified atmosphere with 5% CO_2_ at 37°C.

### 2.3. Fingerprint Analysis by HPLC

For analyzing HPLC fingerprint profiles, SAM (10mg/ml) or the marker glycyrrhizic acid, licorice, and forsythiaside A (10 *μ*L) were injected directly into the Prominence UFLC instrument (Shimadzu LC-10A) with a C-18 reverse phase column. Glycyrrhizic acid, licorice, and forsythiaside A were purchased from Sigma-Aldrich Chemical Co. (St. Louis, Mo, USA). Separation was conducted with a gradient elution of 0.6% phosphoric acid and acetonitrile (0-30 min, B 98%→70%; 30-60 min, B 70%→39%) at a flow rate of 0.8 mL/min. Chromatographic peaks were detected at 260 nm with a SPD-M20A DAD detector. Chromatographic data were collected and processed by an Empower™ chromatographic working station.

### 2.4. Mouse Experiments

The BALB/c mice were obtained from the Experimental Animal Center of the Fourth Military Medical University. The animals were housed under a 12-h light-dark cycle and temperature was kept at 25°C.

For evaluation of the survival and clinical manifestation, groups of neonatal BALB/c mice were inoculated with 100 *μ*l of CA16 (2×10^6^ TCID50) via the i.p. route. In control group, mice were inoculated with 100 *μ*l of PBS via the same route. After inoculation, the mice were monitored daily, and all clinical symptoms were observed and recorded for 15 days. Clinical scores were recorded as: 0 represents healthy; 1 represents reduced mobility; 2 represents limb weakness; 3 represents paralysis; 4 represents death. Body weight, activity, and the occurrence of limb paralysis, morbidity, and death were recorded postinfection.

To evaluate antiviral activity, one-day-old neonatal BALB/c mice were randomly allocated to 6 groups: (1) control group, healthy throughout the experiments, n=15; (2) model group, treated with the same volume of saline after infection, n=8; (3) SAM treatment groups, mice being given various doses (0.8, 1.6, and 3.2 mg/kg) of SAM once by i.p. injection from one day to seven days after infection, n=9 in 0.8 mg/kg, n=11 in 1.6 mg/kg, and n=12 in 3.2 mg/kg; (4) positive control group, mice being given ribavirin by i.p. injection from one day to seven days after infection, n=12.

### 2.5. Ethics Statement

All animal experiments were designed as the direction of the principles expressed in the “The Guidance to Experimental Animal Welfare and Ethical Treatment” by the Ministry of Science and Technology of the People's Republic of China (The Guidance to Experimental Animal Welfare and Ethical Treatment, 2006) and “Guide for the Care and Use of Laboratory Animals” by the National Research Council of the National Academies (National Academy of Science, 2011). The experimental protocol was approved by the Ethics Committee for Animal Experimentation of the Fourth Military Medical University. Animals were handled, ethically treated, and humanly killed as per the rules and instructions of the Ethical Committee. All studies were performed with the approval of the Institutional Ethical Committee (approval number: XJYYLL-2015351).

We have described the possibility of animal death in our study protocol when the study was submitted to our institutional animal ethics committee, and our ethics committee specifically reviewed and approved the mortality aspects of the protocol. In the survival study, humane endpoints were considered but we could not use them because of the pathogenesis of this disease should be recorded. To minimize animal suffering and distress, aspirin, a pain reliever, was given in compliance with the guidelines of the Institute of Medical Biology (IMB), Chinese Academy of Medicine Science (CAMS). The housing conditions, experimental procedures, and animal welfare were in accordance with the local laws and guidelines on the use of laboratory animals. At the end of the study, all of the mice in the experimental and control groups were euthanized via an overdose of anesthesia (pentobarbital sodium).

### 2.6. Cell Viability Assay

For cell viability, SH-SY5Y cells were cultured on 96-well plates overnight. Medium containing 0–97 mg/mL of SAM was added and incubated for 24 h, followed by incubation with MTT (0.5 mg/mL) for another 4 h. The survival rate of cells was expressed as the ratio of optical density (OD) at 490 nm of treated cells to OD_490_ of untreated cells. The data represented the means ± SD of three independent experiments. Cytotoxic concentration of 50% toxic effect (CC50) was defined as the concentration of drug to reduce the viable cell by 50% relative to the untreated control cells.

### 2.7. ELISA

Serum or culture supernatants were collected and saved at -20°C until being used. ELISA for IL-6, caspase 3, and CCL2 were used to measure the anti-inflammation and antiapoptosis activity of SAM. Operating steps were according to the manufacturer's protocol.

### 2.8. Immunoblotting Assays

Total proteins extracts of different cell treatment group and brain tissue (30ug) were mixed with 2x SDS-PAGE sample buffer, boiled for 10 min, and then resolved by 10% SDS-PAGE being transferred onto polyvinylidene difluoride (Millipore, Billerica, MA, USA) membranes. Blots were blocked with 5% skim milk 37°C for 60 min and then reacted with properly diluted monoclonal antibodies (1:1000) including STAT3, PERK, P-PERK, elF2*α*, P- elF2*α*, NF-kB, GAPDH, OASIS, and CHOP at 4°C overnight. Following washing, the membranes were incubated with peroxidase-linked goat anti-rabbit IgG secondary antibody (1:1,000; Santa Cruz Biotechnology) for 1 h 37°C. Protein bands were detected using horseradish peroxidase-conjugated goat anti-mouse IgG antibodies followed by enhanced chemiluminescence reaction (Pierce Biotechnology, USA).

### 2.9. Reverse Transcription and Quantitative Polymerase Chain Reaction (RT-qPCR) Analysis

After the mice were anesthetized, blood were collected; then perfusion needle was inserted through the left ventricle and infused with precooled 0.1 M phosphate-buffered saline (PBS) to wash off the blood for 10min. Then, tissues (brain, heart, skeletal muscle, and lungs) were collected. Then total RNA was extracted by using TRIzol (Invitrogen, USA) according to the standard protocol. 1 mg total RNA with oligo dT primer (forward 5′-ATCCAGTAAGGATCCCAGACT-3′ and reverse 5′-GATTTGCATAGTGGAGAGCAG-3′) and SuperScript III reverse transcriptase kit (Invitrogen) were used to synthesize cDNA as per the manufacturer's instructions. The resultant cDNA was used for real-time PCR with a SYBR Premix Ex Taq TM kit (TaKaRa) and primers. Real-time PCR reactions were carried out for 40 cycles, comprising 95°C for 1 min, 95°C for 15 s, and 60°C for 30 s in a 7900HT Fast Real-Time PCR System (Applied Biosystems). The comparative threshold cycle (Ct) calculation was used to determine the relative gene expression levels, normalized to the internal control (GAPDH).

### 2.10. Transfections

To interfere with the expression of PERK, SH-SY5Y cells were transfected with the siRNA (15 nM) using Lipofectamine RNA interference (RNAi) Max (Life Technologies) according to the manufacturer's instructions. 48 hours posttransfection, cells were used for experiments.

### 2.11. Statistical Analysis

The statistical data are presented as the means ± standard error. All experiments were repeated three times. The differences between two datasets were evaluated using Student's* t*-test with SPSS 18.0 statistical software (SPSS, USA). One-way analysis of variance (ANOVA) was used to compare the difference between more than two datasets. P values of <0.05 were considered to indicate a statistically significant difference.

## 3. Results

### 3.1. SAM Protected Mice from CV16 Infection

We first examined whether SAM has acute toxic effects on BALB/c mice. SAM at a dose of 32g/kg (tenfold of the common dosage) was given to mice for 15 d, and the acute toxicity of the drug was observed. There were no death, no diet, activities, mental state and behavior abnormalities, and no stray, vertical hair phenomenon. These results demonstrated that SAM at 32g/kg has no direct toxic effects on BALB/c mice, so 0.8, 1.6, and 3.2 g/kg SAM were chosen to the further studies.

**Figure 1:**
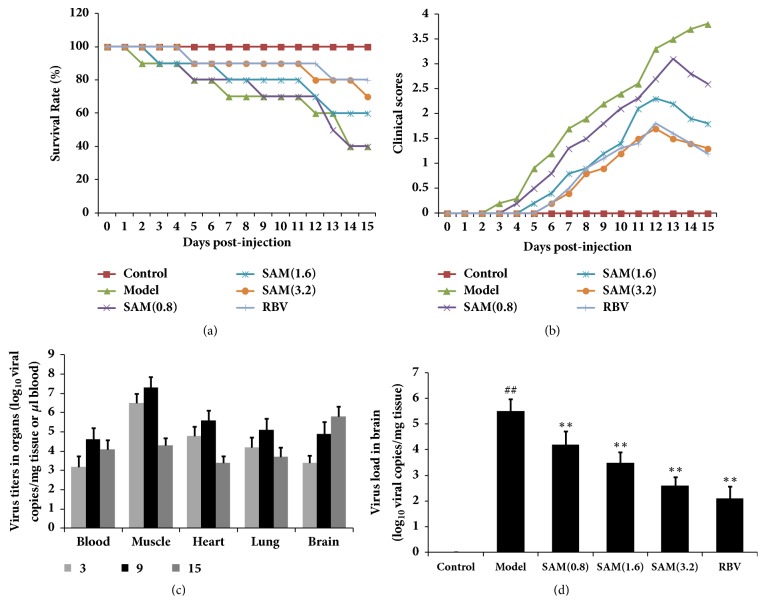
Antiviral activity evaluation of SAM against CVA16 infection in mice. Different groups of 5-day-old BALB/c mice were inoculated i.p. with 2×10^6^ TCID50 of CA16 or with PBS. The inoculated mice were monitored daily for (a) survival and (b) clinical scores. Clinical scores were graded as follows: 0, healthy; 1, reduced mobility; 2, limb weakness; 3, paralysis; 4, death. (c) The virus load in the brain, blood, heart, skeletal muscle, and lungs was measured by real-time PCR. Results are expressed as viral RNA copies/mg tissue or *μ*l blood. (d) Effects of SAM on virus load in brain. Results are expressed as viral RNA copies/mg tissue.^ ##^P<0.01 versus control group; ^∗∗^P<0.01 versus model group.

To observe the effect of SAM on CA16-infected mice, clinical signs including reduced mobility, limb weakness, and paralysis were recorded during the process. As shown in Table 1, mice in CA16 treated group showed reduced mobility, limb weakness, and paralysis, and 60% of them died. In SAM treated groups, these signs were significantly changed, and the mortality was significantly decreased in SAM at dosage of 1.6 and 3.2 g/kg treatment groups. CA16 infected mice started to die at 2 days postinfection and the survival rate was only 40% at 15 days (Figure 1(a)). In SAM treated groups, the survival rates were significantly increased in 1.6 and 3.2 g/kg treatment groups and RBV group. In Figure 1(b), we also found that SAM could reduce the clinical scores at the beginning and delay disease development.

**Figure 2:**
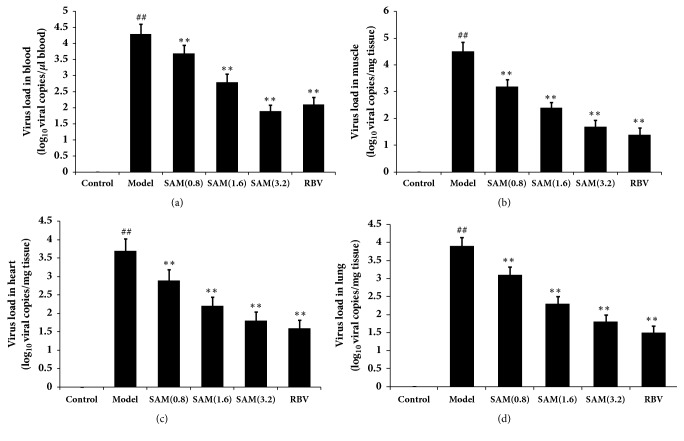
Effects of SAM on virus load in blood, muscle, heart, and lung. The virus load in the blood, heart, skeletal muscle, and lungs was measured by real-time PCR. (a) Virus load in blood. (b) Virus load in muscle. (c) Virus load in heart. (d) Virus load in lung. Results are expressed as viral RNA copies/mg tissue or *μ*l blood. ^##^P<0.01 versus control group; ^∗∗^P<0.01 versus model group.

To detect how CA16 spreads, the virus load in the brain, blood, heart, skeletal muscle, liver, and lungs was measured by real-time PCR. As shown in Figure 1(c), CA16 virus was detected in all the organs/tissues at an early stage of infection (3 days). At 3 and 9 days, the highest virus titer was detected in the skeletal muscles, indicating that the major site of early viral replication was skeletal muscle. These evidences could explain the reason of the early symptom, including reduced mobility, limb weakness, and paralysis. In general, virus load showed a decreasing trend in all organs/tissues from 9 to 15 days except the brain. Indeed, CA16 viral RNA in the brain at 15 days increased ten times higher as compared to that at 9 days, suggesting that, at a relatively late stage of infection, the virus spread to and replicated in the brain. However, important organs infection may be the main causes of death. In Figure 1(d), we found that SAM could significantly decrease the level of CA16 virus in the brain in dose-dependent manners, and the effects of SAM were as good as RBV. Treatment with SAM also significantly decreased the level of CA16 virus in the blood, muscle, heart, and lung (Figure 2).

### 3.2. SAM Inhibited CA16 Induced Apoptosis in Brain

**Figure 3:**
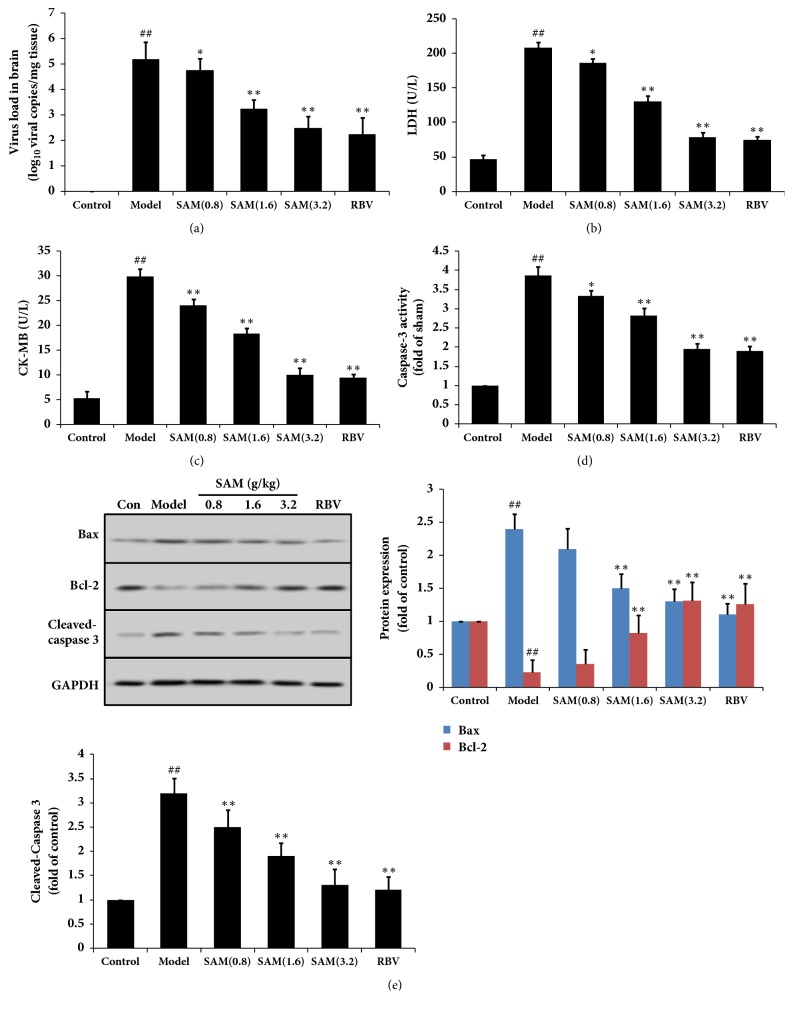
Effect of SAM on apoptosis in brain subjected to CA16 infection. Different groups of 1-day-old BALB/c mice were inoculated i.p. with 2×10^6^ TCID50 of CA16 or with PBS, then SAM were i.p. given for 15 days. (a) Effects of SAM on virus load in brain. LDH (b), CK-MB (c), and caspase 3 levels (d) in serum were detected by the corresponding kits according to the instructions. (e) Cleaved-caspase 3, Bax, and Bcl-2 expression in brain were measured by Western blotting. ^##^P<0.01 versus control group; ^∗^P<0.05; ^∗∗^P<0.01 versus model group.

To determine the mechanism of SAM, apoptosis in brain was measured. The infection in each experiment/group of mice was by verified the virus load in brain (Figure 3(a)). LDH and CK-MB were two commonly used indexes clinically, and changes of their levels indicated injury of the tissues. As shown in the results in Figures 3(b) and 3(c), LDH and CK-MB levels in serum were significantly increased after CA16 infection, indicating that CA16 induced injury in mice. After treatment with SAM or RBV, their levels were significantly decreased and in a dose-dependent manner. We also found that CA16 increased the levels of caspase 3 and the expression of Bax, but inhibited the expression of Bcl-2 (Figures 3(d) and 3(e)). These results demonstrated that CA16 caused apoptosis in brain tissue. However, these changes were reversed by SAM, including decreasing the levels of caspase 3 and Bax and increasing the levels of Bcl-2.

### 3.3. SAM Inhibited Inflammatory Reaction in CA16 Infected Mice

**Figure 4:**
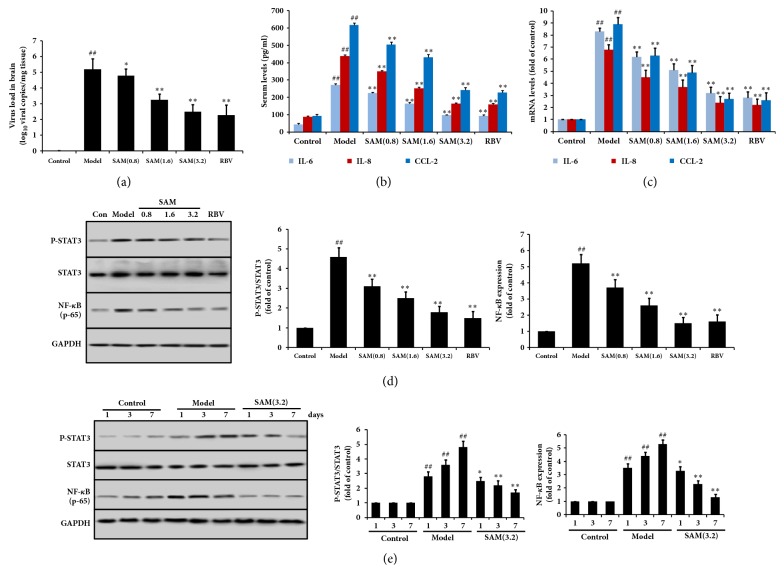
Effect of SAM on inflammatory reaction in brain subjected to CA16 infection. (a) Effects of SAM on virus load in brain. (b) IL-6, IL-8, and CCL-2 levels in serum were detected by the corresponding kits according to the instructions. (b) IL-6, IL-8, and CCL-2 mRNA levels in brain tissues were detected by RT-PCR. (d) P-STAT3, STAT3, and NF-*κ*B were detected in brain tissues by Western blotting. (e) P-STAT3, STAT3, and NF-*κ*B in brain tissues after infection with CA16 for 1, 3, and 7 days. ^##^P<0.01 versus control group; ^∗^P<0.05; ^∗∗^P<0.01 versus model group.

To determine whether SAM has effects on inflammatory reaction induced by CA16, IL-6, IL-8, and CCL-2 levels in serum and brain tissue were measured. The infection in each experiment/group of mice was by verified with the virus load in brain (Figure 4(a)). As shown in Figure 4(b), serum levels of IL-6, IL-8, and CCL-2 were increased significantly in model group (P<0.01), which was compared with control group. After treatment with SAM or RBV, IL-6, IL-8, and CCL-2 levels were decreased significantly and in a dose-dependent manner. And the mRNA levels of IL-6, IL-8, and CCL-2 in brain tissue were also increased by CA16 infection, and SAM decreased them to some extent (Figure 4(c)). The results of Western blotting showed that CA16 induced the phosphorylation of STAT3 and the expression of NF-*κ*B, and SAM significantly decreased P-STAT3 and NF-*κ*B in a dose-dependent manner (Figure 4(d)) and time-dependent manner (Figure 4(e)). These results demonstrated that CA16 induced inflammation in mice, and SAM has protective effect on this inflammatory reaction.

### 3.4. SAM Inhibited CA16 Induced ER Stress in Brain

**Figure 5:**
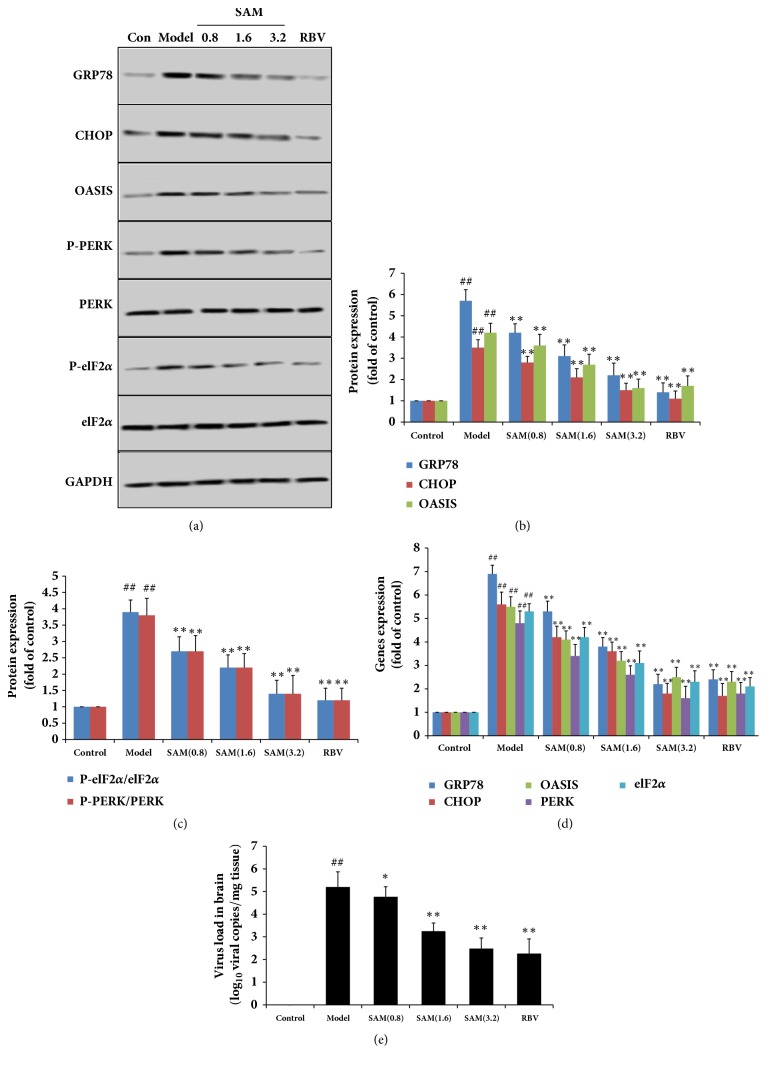
Effect of SAM on ER stress in brain subjected to CA16 infection. Different groups of 1-day-old BALB/c mice were inoculated i.p. with 2×10^6^ TCID50 of CA16 or with PBS, then SAM were i.p. given for 15 days. CHOP, GRP78, PERK, OASIS, and elF2*α* expression levels were detected by Western blotting with the corresponding antibodies. (b) and (c) were the statistical results from (a). (d) mRNA levels of CHOP, GRP78, P-PERK, OASIS, and P-elF2*α* in brain tissues were detected by RT-PCR. (e) Effects of SAM on virus load in brain. ^##^P<0.01 versus control group; ^∗^P<0.05; ^∗∗^P<0.01 versus model group.

ER stress has been observed in the central nervous system of HFMD patients and in mice during CA16 infection. Many literatures have demonstrated that neurons and other related cell lines are vulnerable to ER stress-induced apoptosis [23–25]. To confirm these previous reports, several ER stress makers including CHOP, GRP78, PERK, OASIS, and elF2*α* were measured by Western blotting. As the results shown in Figure 5(a), expressions of CHOP, GRP78, and OASIS were significantly increased in brain of CA16 infected mice, and the phosphorylation of PERK and elF2*α* was also increased. These results indicated the presence of ER stress in the brain during CA16 infection. After treatment with SAM, expressions of CHOP, GRP78, PERK, OASIS, and PERK and elF2*α* phosphorylation were significantly decreased in a dose-dependent manner (P<0.05), confirming that SAM had protective effect on ER stress induced by CA16. The results of mRNA detection were in line with the results of Western blotting (Figure 5(d)). The infection in each experiment/group of mice was verified by the virus load in brain (Figure 5(e)).

### 3.5. SAM Inhibited CA16 Induced Cell Injury In Vitro

**Figure 6:**
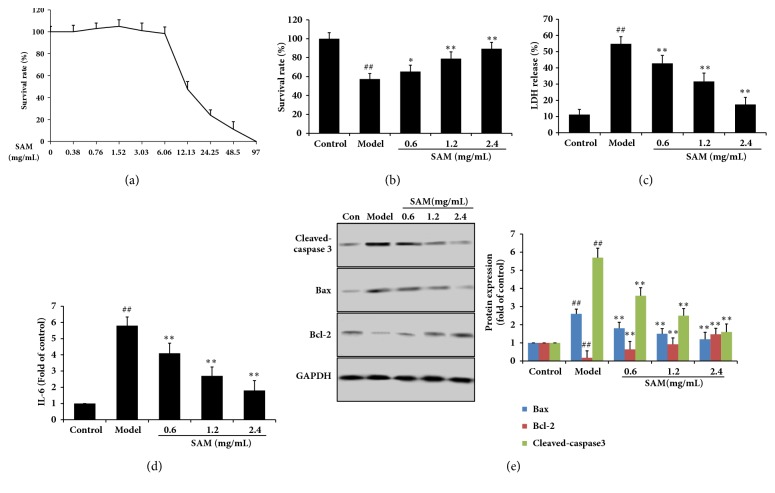
Effect of SAM on CA16 induced cell injury in SH-SY5Y cell. (a) 0–97 mg/mL SAM were given to SH-SY5Y cell for 24 h, and survival rate was detected by MTT. (b) CV16 at the multiplicity of infection (MOI) of 0.5 was used to infect SH-SY5Y cell in the absence or presence of SAM at 0.6, 1.2, or 2.4 mg/mL; survival rate was detected by MTT. (c) LDH was measured by commercial kit and the results were shown as LDH release ratio. IL-6 level was measured by commercial kit as the introduction directed. (e) Cleaved-caspase 3, Bax, and Bcl-2 expression in SH-SY5Y cell were measured by Western blotting. ^##^P<0.01 versus control group; ^∗^P<0.05; ^∗∗^P<0.01 versus model group.

To evaluate the cytotoxicity of SAM, SH-SY5Y cells were treated with the concentration range of 0–97 mg/mL. In vitro cytotoxicity assay showed that SAM was not cytotoxic to SH-SY5Y in the concentration range of 0-3 mg/mL after pretreatment for 24 h (Figure 6(a)). 50% cytotoxicity concentration (CC 50) of SAM was 9.59 mg/mL, so 0.6, 1.2, and 2.4 mg/mL SAM were selected for further studies. As shown in Figure 6(b), CA16 caused significant cell death in model group (P<0.05), which is compared with the control group. After treatment with SAM, the survival rates of SH-SY5Y cell were dose-dependently increased. CA16 infection also induced the release of LDH and IL-6, which were the marker of cell injury (Figures 6(c) and 6(d)) and caused cell apoptosis in cells (Figure 6(e)). SAM decreased the levels of IL-6 and LDH and expression of cleaved-caspase 3 and Bax; it however increased the expression of Bcl-2. These results demonstrated that SAM protected SH-SY5Y cell from CA16 induced cell injury in vitro.

### 3.6. SAM Inhibited CA16 Induced ER Stress and Inflammatory Response In Vitro

**Figure 7:**
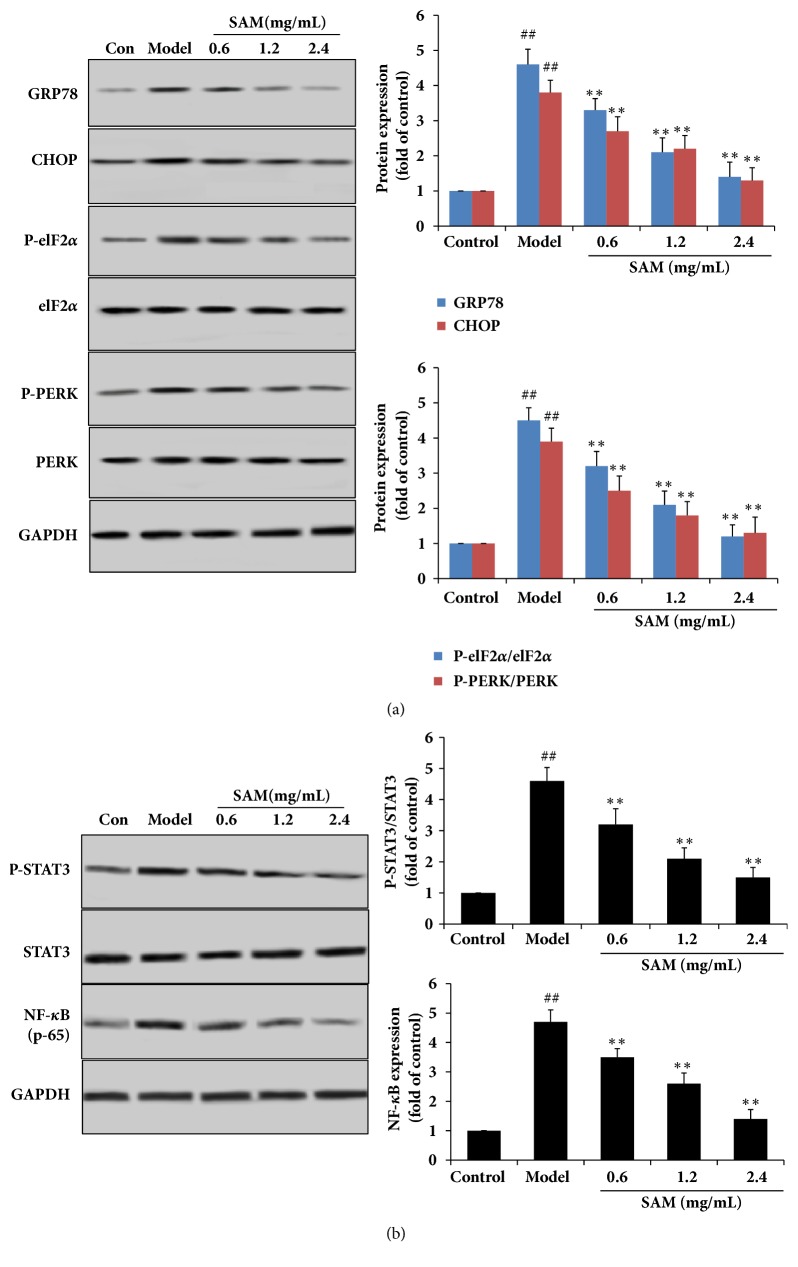
Effect of SAM on CA16 induced ER stress and inflammatory response in SH-SY5Y cell. (a) After different treatment, total proteins were extracted. CHOP, GRP78, PERK, P-PERK, OASIS P- elF2*α*, and elF2*α* expression levels were detected by Western blotting with the corresponding antibodies. (b) CA16 induced expression of NF-*κ*B and phosphorylation of STAT3, and SAM treatment significantly decreased this effect. ^##^P<0.01 versus control group; ^∗^P<0.05; ^∗∗^P<0.01 versus model group.

To determine whether CA16 induces ER stress in vitro, markers of ER stress including PERK, CHOP, GRP78, and elF2*α* were detected. As shown in Western blotting results in Figure 7(a), CA16 increased the expression of CHOP and GRP78 and phosphorylation levels of PERK and elF2*α*, indicating that CA16 induced ER stress in vitro. After treatment with SAM for 24h, the expression of CHOP and GRP78 and phosphorylation levels of PERK and elF2*α* were significantly decreased in a dose-dependent manner (P<0.05). STAT3 and NF-*κ*B were also detected to test the possible inflammation related signaling pathway. In Figure 7(b), we could find that CA16 treatment significantly increased the phosphorylation level of STAT3 and the expression of NF-*κ*B, and these results were in accordance with the results in vivo. SAM dose-dependently decreased the phosphorylation level of STAT3 and the expression of NF-*κ*B, indicating that the inhibiting effect of SAM might be through inhibiting the STAT3/ NF-*κ*B pathway.

### 3.7. SAM Inhibited the Inflammatory Response through the PERK/STAT3/ NF-*κ*B Pathway

**Figure 8:**
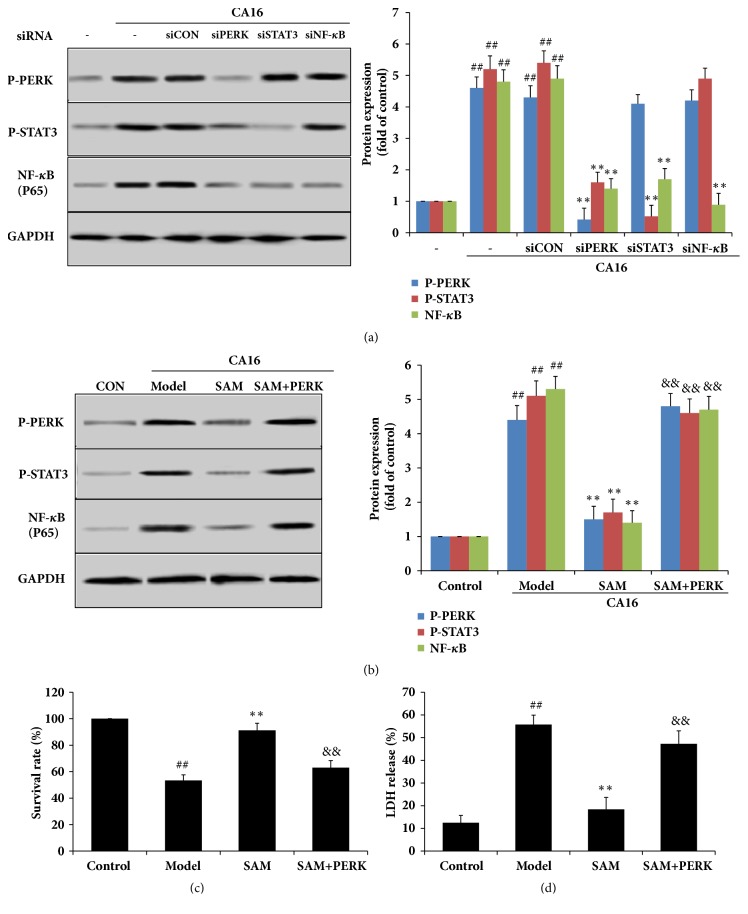
SAM inhibited the inflammatory response through the PERK/STAT3/ NF-*κ*B pathway. (a) SH-SY5Y cells were transfected with PERK, STAT3, and NF-*κ*B-specific siRNA (15 nM), respectively, and then subjected CA16 infection. Total proteins were extracted and PERK, P-PERK, NF-*κ*B(p-65), P-elF2*α*, and elF2*α* expression levels were detected by Western blotting with the corresponding antibodies. ^##^P<0.01 versus control group; ^∗^P<0.05; ^∗∗^P<0.01 versus siCON group. (b) SH-SY5Y cells were overexpressed PERK with PERK lentiviral activation particles as the protocol direction and then subjected to CA16 infection and treated with SAM (2.4 mg/mL). PERK, P-PERK, NF-*κ*B(p-65), P-elF2*α*, and elF2*α* expression levels were detected by Western blotting; survival rate and LDH release rate were also detected by kits. ^##^P<0.01 versus control group; ^∗^P<0.05; ^∗∗^P<0.01 versus model group; ^&&^P<0.01 versus SAM treatment group.

To make the relationship between PERK ER stress pathway and STAT3/NF-*κ*B inflammation pathway clear, we determined the effect of PERK, STAT3, and NF-*κ*B siRNA transfection on other proteins expression. In Figure 8(a), PERK-siRNA transfection significantly decreased the phosphorylation level of STAT3 and the expression of NF-*κ*B induced by CA16 infection. STAT3-siRNA transfection significantly decreased the phosphorylation level of STAT3 and the expression of NF-*κ*B induced by CA16 infection, but has no effect on PERK expression. NF-*κ*B-siRNA transfection significantly decreased the expression of NF-*κ*B induced by CA16 infection, but had no effect on PERK expression and STAT3 phosphorylation. A scrambled RNA transfection was used as a negative control in this study. These results demonstrated that CA16 induced ER stress through activating PERK pathway and then phosphorylated STAT3 protein and promoted NF-*κ*B expression, thus inducing inflammation in SH-SY5Y cell.

We next sought to determine if the protective effect of SAM was dependent on inhibiting PERK/STAT3/NF-*κ*B signaling. PERK was overexpressed in the future studies. After SAM treatment, PERK expression was significantly lower, and also STAT3 phosphorylation and NF-*κ*B expression were lower. However, overexpression of PERK caused the increase of cytosolic PERK expression under CA16 infection, phosphorylated STAT3 protein, and promoted NF-*κ*B expression. SAM could protect cells from CA16 induced cell injury under normal condition, once PERK overexpression, the cell death, and IL-6 levels were significantly increased. The protective effects of SAM were abolished by PERK overexpression (Figures 8(b)–8(d)).

### 3.8. Fingerprint Profiling of SAM

**Figure 9:**
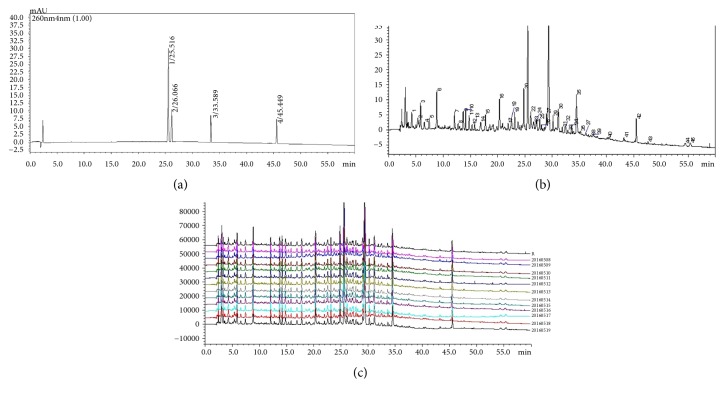
HPLC fingerprints profiles SAM and marker components. Marker components of forsythoside A, liquiritin, forsythin, and glycyrrhizic acid (a) and SAM (b) were analyzed using HPLC with a C-18 reverse phase column. Eluents were detected at 260 nm. (b) HPLC fingerprints of 12 batches of SAM (similarity was more than 0.998).

Liquid chromatography can always be common used as a tool to measure drugs contents and developed for the quality control of traditional Chinese medicine. To determine the fingerprint of SAM, SAM and marker components of forsythoside A, forsythin, glycyrrhizic acid, and liquiritin as well were analyzed using HPLC with a C-18 reverse phase column (Figure 9). As shown in Figure 9(b), the retention time of marker components was in accordance with the single chemical compound at 260 nm, and chromatographic peaks marked with 26, 27, 39, and 41 were forsythoside A, liquiritin, forsythin, and glycyrrhizic acid, which correspond to the positions in Figure 9(a). Next, we analyzed the 12 batches of SAM samples. The HPLC fingerprinting profiles were shown in Figure 8(c), and 42 common peaks were found. The similarity between the values obtained for the 12 samples was higher than 0.998, indicating a high consistency and stability between the batches of SAM tested. The average amounts of forsythoside A, liquiritin, forsythin, and glycyrrhizic acid in 12 batches of SAM were 1.85, 0.07, 0.06, and 0.25 mg•ml^−1^, and RSD were 1.10%, 0.98%, 1.21%, and 0.84%, respectively.

## 4. Discussion

In the Asia-Pacific region, HFMD has been serious public health problems. CA16 and EV71 are two main causative viruses which are both responsible for the disease becoming widespread [26, 27]. However, there are a few available drug treatments and vaccines to treat CVA16 or EV71 infections effectively. Since EV71 was initially reported to be associated with severe complications in some regions, most of antiviral and vaccine studies have been focusing on it [28]. Indeed, CA16 can also cause severe complications, even death, and, in Mainland China, it is responsible for nearly 50% of all HFMD cases [29]. With increasing public concern about CA16 infections, investigation of the pathogenesis of CA16 and development of the efficient drugs seem more necessary. Siji Antiviral Mixture (SAM) has been used as antivirus infection drug clinically for many years, and it showed very good therapeutic effects on treating viral cold, influenza, and mumps. In recent years, it was also used in treating HFMD and had very good clinical effects. However, the mechanism and main ingredients were largely unknown. In this study, neonatal mice and SH-SY5Y cells were used to establish an infection model, and the therapeutic effects were evaluated.

In present study, we found that SAM has no acute toxic effects on BALB/c mice up to 32g/kg, which is tenfold the common dosage clinically, so SAM at the dosage of 0.8, 1.6, and 3.2 g/kg was chosen for further studies. SAM treatment significantly improved the clinical signs, which include reduced mobility, limb weakness, paralysis, and death induced by CA16 infection in mice. At the initial stage of viral replication (3 to 9 days), the highest virus content was detected in the skeletal muscles of infected mice. At the last stage of viral replication (9 to 15 days), CA16 viral RNA in the other organs showed a decreasing trend; however, in brain, CA16 viral RNA increased ten times (Figure 1). These results demonstrated that CA16 causes cranial nerve lesion at last stage, and this might be the main death effect, so this study was focusing on the CA16 induced cranial nerve lesion and the nerve cell protection of SAM was investigated. The results in Figure 1(d) showed that SAM inhibited viral replication in brain, indicating potential cerebral protection effect.

Many literatures have reported that many viruses could modulate the cell cycle to increase their replication efficiency and cause host cell apoptosis [30]. CA16 infection significantly increased protein expression of Bax, a proapoptotic protein during the early phase of apoptosis, further indicating that CA16 infection leads to cell apoptosis [31]. LDH and CK-MB were two cytokines in common body, and their levels were low in healthy body, but when the body was injured by stimulations or virus infections, leading to cell membrane breakage, they are released to the blood. Thus, their levels were always used as diagnosis index clinically. In this study, CA16 infection increased the levels of LDH and CK-MB, indicating CA16 caused cell death in mice. The levels of caspase 3 and Bax were also increased, and Bcl-2 was decreased by CA16 infection, indicating apoptosis was induced in brain. SAM treatment decreased the levels of LDH, CK-MB, caspase 3, and Bax and increased Bcl-2 expression, showing the antiapoptosis effects of SAM.

Endoplasmic reticulum (ER) stress and inflammation which frequently involve misfolded and aggregated proteins are particularly relevant to neurodegenerative diseases [32]. Moreover, ER stress is important in neuroinflammatory diseases. Once cells were infected virally, the synthesis of the viral polypeptides and the replication of the viral genome induce ER stress in mammalian cells [33]. To restrict the replication and spread of the virus, the cell may undergo autophagy-dependent cell death or apoptosis when the infection eventually endangers the cells. Thus, protecting cells from CA16 induced ER stress and inflammation might be effective. Proinflammatory cytokines, including IL-6, IL-8, and CCL-2 levels, were increased at serum and mRNA levels in this study (Figures 4(a) and 4(b)). SH-SY5Y cell directly treated with CA16 also showed the high levels of IL-6. These results indicated that inflammation was induced in mice by CA16 infection. GRP78 is an ER-resident chaperone and a master regulator of ER stress [34, 35]. ER-stress-induced apoptosis is mediated largely by CHOP, a transcription factor that is homologous to C/EBP (CCAAT/enhancer-binding protein) and is downstream of the PERK–eIF2*α*–ATF4 pathway [32]. The expression of GRP78 and CHOP, and phosphorylation levels of PERK and eIF2*α* are most commonly used to evaluate ER stress in cells [36]. Here, we found that the expression of GRP78 and CHOP and phosphorylation levels of PERK and eIF2*α* were increased following CA16 infection in mice and SH-SY5Y cell, which suggests the occurrence of ER stress. Treatment with SAM significantly decreased the levels of proinflammatory cytokines and the expression of ER stress markers.

NF-*κ*B, a key transcriptional regulator, plays an important role in the onset of inflammation. Under normal conditions, NF-*κ*B binds to a member of the family of inhibitors of NF-*κ*B (I*κ*B) and remains in an inactive state. When cells are subjected to inflammatory stimuli, I*κ*B was phosphorylated and subsequently degraded, and then NF-*κ*B expression was increased and translocated to the nucleus, where it induces numerous inflammatory genes expression [37, 38]. During viral infection, ER protein-folding load increasing has been shown to result in the activation of NF-*κ*B [39]. Previous study had shown that JAK/STAT activation is a primary response to ER stress and subsequent inflammatory gene expression [40]. When starved cancer cells undergo ER stress, NF-*κ*B and STAT3 work together to drive IL-6 expression [41]. However, the interaction between NF-*κ*B and STAT3 and the relationships between ER stress and STAT3 undergoing CA16 infection in brain were largely unknown. In the current study, we found that CA16 infection significantly increased the phosphorylation level of STAT3 and the expression of NF-*κ*B, together with the increase of PERK expression. We also found that the activation of STAT3 and NF-*κ*B by CA16 infection in PERK-siRNA transfected cells was significantly inhibited. In STAT3-siRNA transfected cells, NF-*κ*B expression was inhibited only. These results demonstrated that CA16 activated PERK pathway firstly, then phosphorylated STAT3 protein, and promoted NF-*κ*B expression, thus inducing inflammation and apoptosis in brain cells. To further study whether the effect of SAM was through PERK/STAT3/NF-*κ*B signaling pathway, PERK was overexpressed. Overexpression of PERK abolished the inhibiting effects of SAM on STAT3 and NF-*κ*B activity and the cerebral protection effects of SAM. Our data pointed toward that PERK /STAT3/NF-*κ*B-dependent pathway driving inflammatory genes expression and cells injury in brain cells in response to CA16 infection. The protective effect of SAM was through inhibiting PERK/STAT3/NF-*κ*B signaling pathway.

Generally, several herbals were contained in an herbal formulation, and, due to differences in plant origins, practices, climate conditions, cultivation areas, and processing protocols among others, the chemical composition of herbal formulations may vary in a large range [42–44]. This may lead to wide disparities in quality and therapeutic effects among different samples. Therefore, it was necessary to develop an effective and feasible method for the quality control of SAM. Thus, we have developed and validated a simple and rapid HPLC method for the simultaneous determination of 42 common peaks in the chemical profile. Forsythoside A, forsythin, glycyrrhizic acid, and liquiritin were identified and confirmed by their standards. Accuracy, high repeatability, recovery, and intraday and interday precision were achieved with this HPLC method in our validation procedure (date not shown). Twelve batches of SAM were homogeneous (similarity higher than 0.998). Thus, our proposed method could improve quality control for SAM.

In conclusion, SAM protected brain cells from CA16 infection induced ER stress and inflammation through inhibiting PERK/STAT3/NF-*κ*B signaling pathway. SAM could be a safe and potential therapeutic agent against CA16 infection. In addition, PERK/ STAT3/NF-*κ*B pathway could be a new therapeutic target to combat neuroinflammation associated with CA16 infection.

## Figures and Tables

**Table 1:** Summary of morbidity and mortality of mice infected with CA16 at different treatments.

**Groups**	**Total no.**	**No. of mice exhibiting symptoms**			**No. of deaths**
		**Reduced mobility **	**Limb weakness **	**Paralysis **	
Control	10	0	0	0	0
Model	10	10(100%)	10(100%)	6(60%)	6(60%)
SAM(0.8g/kg)	10	10(100%)	9(90%)	4(40%)	6(60%)
SAM(1.6g/kg)	10	8(80%)	7(70%)	3(30%)	4(40%)
SAM(3.2g/kg)	10	7(70%)	5(50%)	1(10%)	3(30%)
RBV(0.01g/kg)	10	7(70%)	4(40%)	1(10%)	2(20%)

## Data Availability

The data used to support the findings of this study are available from the corresponding author upon request.
